# Impact of the timing of initiation of a smartphone-based application on very short-term postoperative satisfaction after total hip arthroplasty: a prospective observational study

**DOI:** 10.1186/s12891-026-09959-8

**Published:** 2026-05-12

**Authors:** Tsutomu Nakayama, Sachiyuki Tsukada, Naoyuki Hirasawa

**Affiliations:** 1 Department of Rehabilitation, Hokusuikai Kinen Hospital, 3-2-1 Higashihara, Mito, Ibaraki 310-0035 Japan; 2 Department of Orthopaedic Surgery, Hokusuikai Kinen Hospital, 3-2-1 Higashihara, Mito, Ibaraki 310-0035 Japan

**Keywords:** Total hip arthroplasty, Smartphone application, Physiotherapy, Patient satisfaction, Digital health

## Abstract

**Background:**

This prospective observational study included patients undergoing primary total hip arthroplasty (THA). Shortened hospital stays after THA have increased the importance of patient self-management and early engagement in rehabilitation. Smartphone-based digital health applications are increasingly used in perioperative care; however, the clinical value of preoperative initiation remains unclear. To examine whether preoperative initiation of a smartphone-based physiotherapy support application improves postoperative patient satisfaction and behavioral outcomes compared with postoperative initiation in patients undergoing primary THA.

**Methods:**

This prospective single-center observational study included patients who underwent primary THA between October 2024 and March 2025. Patients were classified into preoperative or postoperative application initiation groups based on the timing of use. The preoperative group started use at a mean of 19.3 ± 6.4 days before surgery (range: 7 to 30 days), while the postoperative group began use on postoperative day 1. Allocation was determined by smartphone ownership, patient preference, and timing of institutional implementation. Those who began application use one day before surgery were excluded. The primary outcome was the dissatisfaction score of the Japanese Orthopaedic Association Hip Disease Evaluation Questionnaire (JHEQ). Secondary outcomes included the Forgotten Joint Score–12 (FJS-12) and stages of behavior change. Outcomes were assessed preoperatively and at 1 week, 1, 3, and 6 months postoperatively.

**Results:**

A total of 137 patients were analyzed. The preoperative initiation group demonstrated a lower JHEQ dissatisfaction score at 1 week postoperatively compared with the postoperative group (mean difference: -10.4, 95% confidence interval (CI): -19.62 to -1.20; unadjusted *p* = 0.03; adjusted *p* = 0.15 after Bonferroni correction). The effect size at 1 week was Cohen’s d = 0.39 (95% CI: 0.04 to 0.74). No significant between-group differences were observed at later time points or in FJS-12 scores. At 1 month, behavior change stage distributions differed between groups (*p* = 0.02).

**Conclusions:**

Earlier (preoperative) initiation of a smartphone-based physiotherapy support application was associated with a clinically relevant reduction in patient dissatisfaction at one week postoperatively and more favorable early behavioral readiness for rehabilitation. Shifting the timing of digital health support to the preoperative phase, thereby facilitating earlier exposure to education and guidance, may enhance the early postoperative patient experience and facilitate engagement. The clinical magnitude of this effect was supported by the effect size and confidence interval at the initial recovery stage.

## Background

Total hip arthroplasty (THA) is an established and effective surgical intervention for patients with end-stage hip disorders, resulting in substantial improvements in pain, physical function, and health-related quality of life [1, 2]. Advances in perioperative management and fast-track protocols have shortened hospital stays, thereby increasing the responsibility placed on patients to manage their own recovery after discharge [3].

Despite favorable surgical outcomes, early postoperative dissatisfaction remains a clinically relevant issue. In the immediate postoperative period, particularly within the first week following THA, patients frequently experience pain, functional limitations, and uncertainty regarding safe activity levels and rehabilitation progress [4]. Consequently, patient-reported outcome measures (PROMs), including satisfaction and joint awareness, have become increasingly important indicators of recovery quality [5].

In addition to physical recovery, psychological and behavioral factors such as readiness for behavioral change and self-efficacy are known to influence adherence to rehabilitation and long-term functional outcomes [6, 7]. The transtheoretical model of behavior change provides a useful framework for evaluating patients’ readiness to engage in health-promoting behaviors, including exercise and self-management [8].

Smartphone-based digital health interventions have emerged as promising tools to support perioperative rehabilitation by providing education, exercise guidance, and continuous communication with healthcare professionals [9–11]. While previous studies have reported benefits of postoperative application use after joint arthroplasty [12, 13], evidence regarding the clinical value of initiating digital support before surgery remains limited, particularly with respect to early postoperative patient satisfaction and behavioral outcomes.

Early postoperative outcomes, particularly within the first week after total hip arthroplasty, have been shown to strongly influence patients’ perceptions of recovery, satisfaction, and adherence to postoperative rehabilitation. This early phase represents a critical period during which patients form expectations regarding their recovery trajectory and engagement in self-management behaviors. Therefore, evaluating interventions that target this early postoperative period is clinically meaningful and aligns with contemporary enhanced recovery strategies.

The purpose of this study was to evaluate the clinical value of preoperative initiation of a smartphone-based physiotherapy support application in patients undergoing primary THA, compared with initiation after surgery. To our knowledge, no prospective study has directly compared preoperative versus postoperative initiation of a physiotherapy support application with respect to early postoperative dissatisfaction after THA.

We hypothesized that patients who initiated application use preoperatively would demonstrate lower dissatisfaction during the early postoperative period, particularly at one week after surgery, compared with patients who initiated application use postoperatively. We further hypothesized that preoperative initiation would be associated with more favorable behavioral change stages during early recovery.

## Materials and methods

### Study design and participants

This prospective single-center observational study was conducted at Hokusuikai Kinen Hospital between October 2024 and March 2025. Patients in both the preoperative and postoperative initiation groups were enrolled concurrently during this study period. This study is reported in accordance with the STROBE guidelines for observational studies. The study design and reporting followed general recommendations for observational research in clinical rehabilitation settings. No investigator-controlled intervention, allocation, or randomization was performed.

Patients who underwent primary THA during the study period were screened for eligibility. Exclusion criteria included THA performed for proximal femoral fracture; contralateral THA, total knee arthroplasty, or unicompartmental knee arthroplasty during the study period; intraoperative or postoperative periprosthetic fracture; perioperative surgical site infection; postoperative peripheral nerve injury; history of central nervous system disorders; severe internal or psychiatric disease; severe cognitive impairment; social hospitalization or discharge requiring non-standard follow-up; and refusal to provide informed consent.

**Fig. 1:**
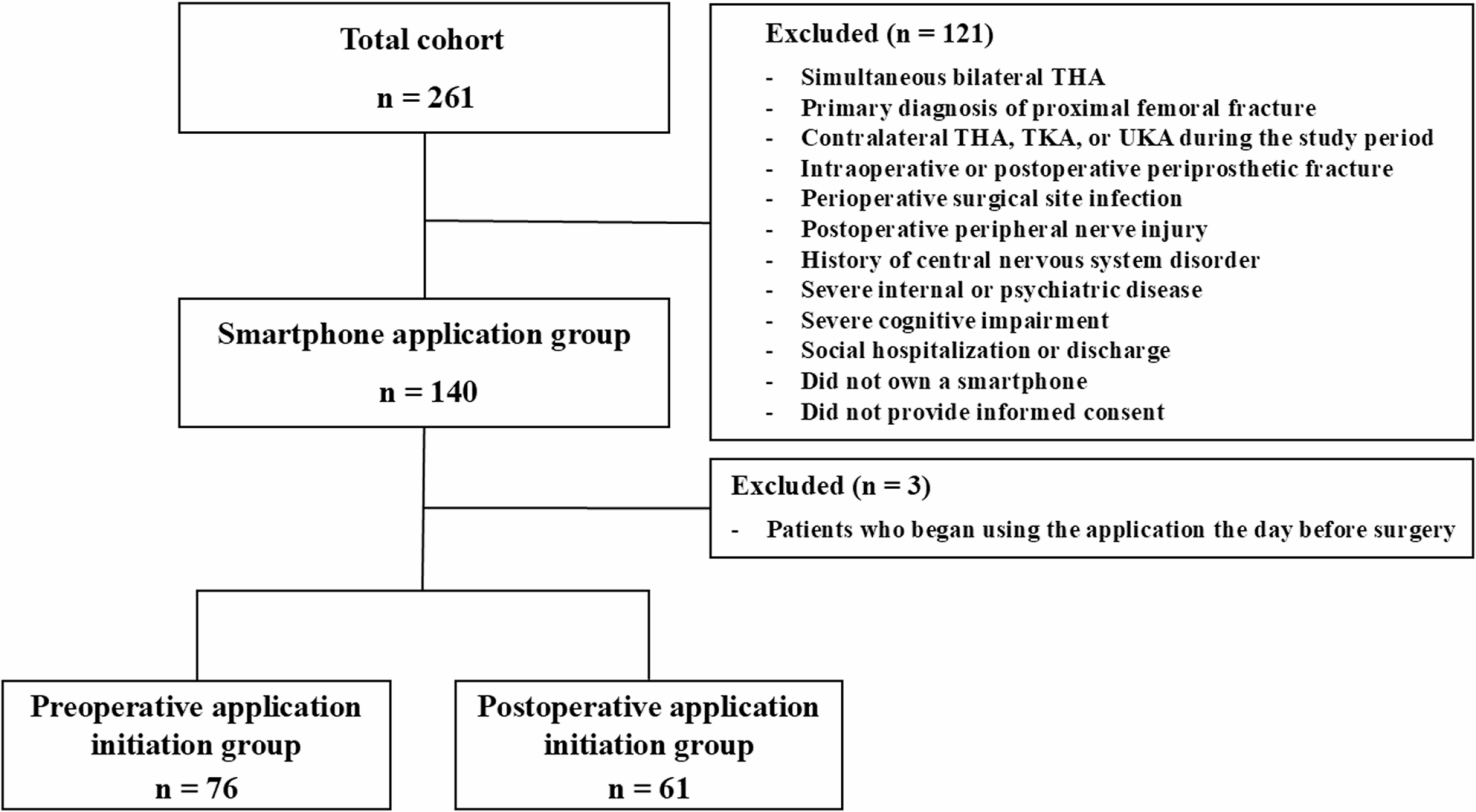
Participant flow diagram. THA, total hip arthroplasty; TKA, total Knee arthroplasty; UKA, unicompartmental knee arthroplasty. Flow diagram illustrating patient selection, exclusion criteria, and group allocation. A total of 261 patients undergoing primary total hip arthroplasty (THA) were screened, and 137 patients were included in the final analysis (Preoperative application initiation group, *n* = 76; Postoperative application initiation group, *n* = 61)

Patients who began application use one day before surgery were excluded from this comparison (Fig. 1).

### Ethical considerations

This study was approved by the Ethics Committee of Hokusuikai Kinen Hospital in December 2023 (approval number: 089).

The smartphone application was introduced as part of routine clinical care at the hospital. Use of the application was determined by the implementation period, smartphone ownership, and the patient’s own preference, and was not assigned or mandated for research purposes. No protocol-driven intervention, allocation, or manipulation of application use was performed by the investigators. The frequency and content of application use were not controlled by the research team.

All participants received a detailed explanation of the study objectives and procedures, and written informed consent was obtained specifically for the use of clinical and questionnaire data for research analysis. The study was conducted in accordance with the Declaration of Helsinki and relevant ethical guidelines for medical research.

### Grouping according to application initiation timing

Patients were classified into a preoperative initiation group and a postoperative initiation group based on the timing of smartphone application initiation. This allocation was determined by the timing of institutional implementation, smartphone ownership, and patient preference. All participants received a preoperative explanation of the application; however, the postoperative group consisted of patients who chose to initiate the application after surgery or were only able to complete the setup during their hospital stay. Patients who began application use one day before surgery were excluded from this comparison. The preoperative initiation group used the application for a mean of 19.3 ± 6.4 days (range, 7 to 30 days) before surgery, whereas all patients in the postoperative initiation group began use on the first postoperative day.

### Surgical procedure and clinical pathway

All THA procedures were performed or supervised by one of three experienced orthopedic surgeons specializing in joint arthroplasty. A minimally invasive anterolateral approach (modified Watson-Jones approach) was used in all cases. A portable navigation system (AR Hip Navigation System; Zimmer Biomet, Japan) was employed to assist implant positioning.

Postoperatively, no restrictions were imposed on joint range of motion or weight bearing. A standardized clinical pathway was applied, including admission on the day before surgery, mobilization on postoperative day 1, and discharge on postoperative day 7. Outpatient face-to-face physiotherapy after discharge was provided as needed.

### Smartphone application intervention

**Fig. 2:**
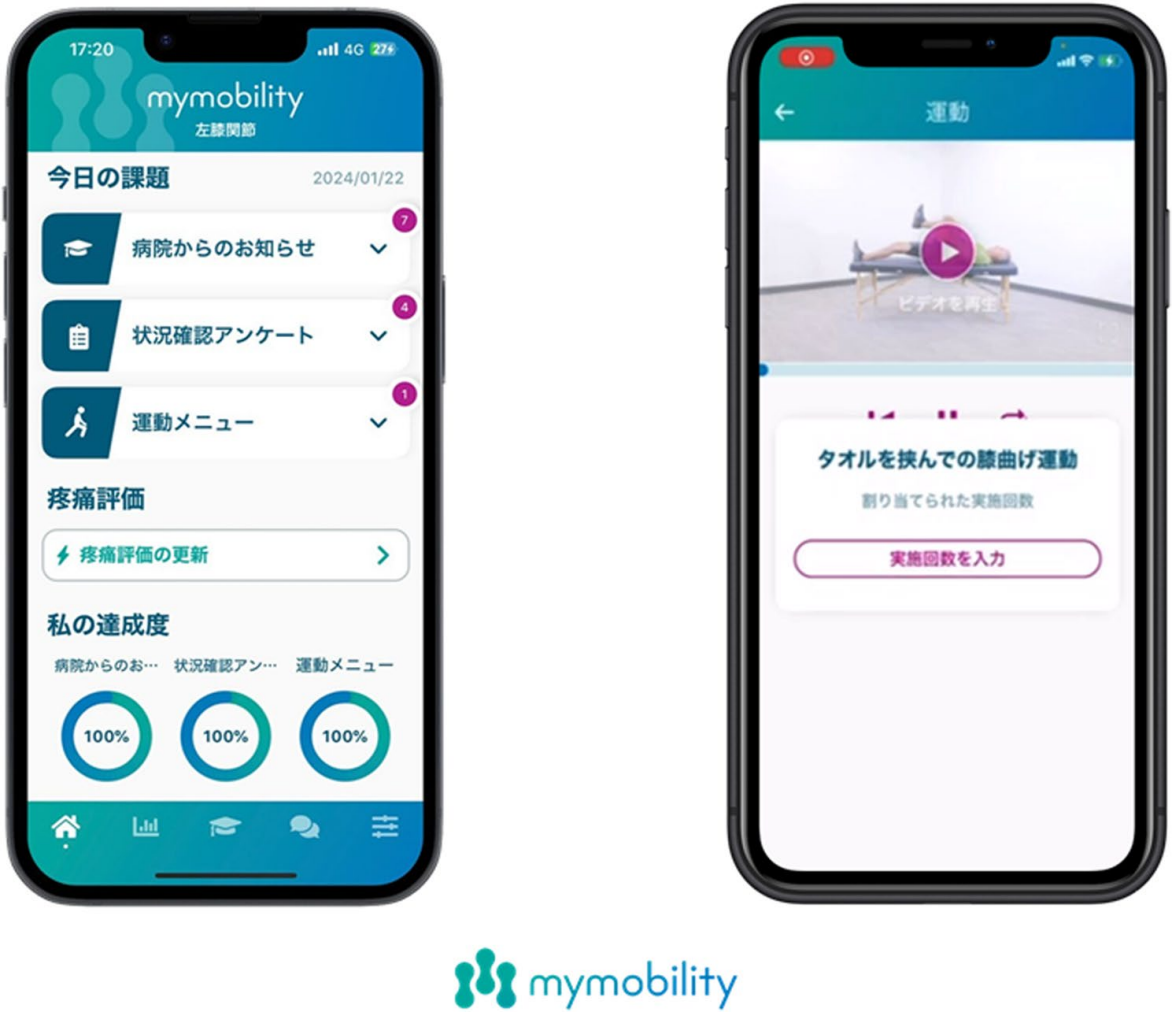
Smartphone application “mymobility”. Representative screenshots of the smartphone-based physiotherapy support application “mymobility,” illustrating its main functions, including exercise guidance, perioperative educational content, and progress tracking for patients undergoing total hip arthroplasty

The smartphone application used in this study was mymobility (Zimmer Biomet, Japan), a commercially available digital health platform designed to support perioperative rehabilitation for orthopedic patients. The application provides individualized exercise programs, educational content, and longitudinal monitoring of symptoms and activity levels to enhance patient engagement (Fig. 2).

The preoperative program delivered via the application consisted of structured exercise and educational modules. The exercise menu, provided through video-based instructions, included muscle strengthening and stretching exercises targeting the periarticular hip muscles (recommended at a low intensity, 3 sets of 20 repetitions per day, 3 times per week). Additionally, patients were encouraged to engage in aerobic exercises, such as walking or aquatic exercises (20 min per day, 3 times per week). The educational component utilized both video and text-based materials to provide guidance on the surgical procedure, potential postoperative complications, hospital life, and the typical postoperative recovery trajectory.

The application also includes a bidirectional messaging function that enables communication between patients and healthcare professionals. This function was available 24 h a day to address patient inquiries during the preoperative and postoperative periods. Messages were primarily managed by physiotherapists and ward nurses, with escalation to orthopedic surgeons when clinically indicated.

The explanation of the application and the obtaining of written informed consent were primarily conducted during the preoperative outpatient visit, approximately one month before surgery. For patients who did not complete this process during the outpatient visit, the explanation and consent were provided on the day of admission (the day before surgery). Application initiation during the preoperative period was at the patient’s discretion. Consequently, participants were divided into those who began using the application preoperatively and those who initiated use on the first postoperative day with support from healthcare staff.

To ensure appropriate use and adherence, clinicians provided face-to-face operational guidance and technical support to all participants at the time of discharge. While the frequency of use was not quantitatively tracked through application logs, this direct support served to reinforce patient engagement with the rehabilitation program. The hospital maintained an institutional subscription, and all costs were covered by the institution. No authors were involved in application development or held any related intellectual property.

### Outcome measures

The primary outcome was patient dissatisfaction assessed using the dissatisfaction domain of the Japanese Orthopaedic Association Hip Disease Evaluation Questionnaire (JHEQ) [14]. This domain consists of a single-item, 100-mm Visual Analogue Scale (VAS) that measures the patient’s current level of satisfaction regarding their hip condition. Patients mark their status on a continuum where the left end (0 mm) represents “complete satisfaction” and the right end (100 mm) represents “complete dissatisfaction.” Secondary outcomes included joint awareness assessed using the Forgotten Joint Score–12 (FJS-12) [15] and behavioral readiness assessed using the behavioral change stage model [6].

Assessments were conducted preoperatively and at 1 week, 1 month, 3 months, and 6 months postoperatively.

This study was designed to evaluate the impact of the timing of initiation of digital health support (i.e., early preoperative exposure to education and guidance) on postoperative outcomes, rather than to assess the intrinsic effectiveness of the digital platform compared to non-digital alternatives.

### Sample size calculation

Sample size estimation was based on the primary outcome of JHEQ dissatisfaction at one week postoperatively. Based on preliminary data from the comprehensive evaluation study and previous reports examining early postoperative satisfaction after THA [5, 16], a between-group difference of approximately 10 points on the JHEQ dissatisfaction scale was considered clinically meaningful. Assuming a standard deviation of 15 points, an alpha level of 0.05, and a power of 80%, a minimum of 36 patients per group was required to detect a statistically significant difference using a two-sample t-test.

Given the observational nature of the study and anticipated exclusions, the available sample size exceeded the minimum required, providing sufficient power for the primary analysis. This calculation was used to confirm adequacy of the available sample rather than to determine enrollment, given the observational design.

### Statistical analysis

Continuous variables were summarized as means and standard deviations, and categorical variables as frequencies and percentages. Baseline between-group comparisons for continuous variables were performed using independent two-sample t-tests, while categorical variables were compared using the chi-square test or Fisher’s exact test, as appropriate.

To account for the longitudinal nature of the data and repeated measurements, we employed the following approaches.

For JHEQ dissatisfaction scores and FJS-12 scores, a two-way repeated-measures Analysis of variance (ANOVA) was performed to examine the effects of group, time, and their interaction. Greenhouse-Geisser corrections were applied when the assumption of sphericity was violated (Mauchly’s test, *p* < 0.05). When a significant interaction or main effect was found, post-hoc between-group comparisons at each time point were conducted using independent t-tests to evaluate the treatment effect at each assessment period. To control for Type I error inflation due to multiple comparisons across the five time points, Bonferroni corrections were applied to the p-values of these post-hoc tests. Effect sizes (Cohen’s d) and their 95% confidence intervals (CI) were also calculated to assess the magnitude of the differences between groups.

For behavioral change stages, which are ordinal categorical data, a cumulative link mixed model (CLMM) was utilized to evaluate the longitudinal impact of the intervention while accounting for individual random effects.

A two-sided *p*-value of < 0.05 was considered statistically significant. Statistical analyses were performed using EZR (version 1.55), a graphical user interface for R (The R Foundation for Statistical Computing, Vienna, Austria) designed for medical statistics [17]. The ‘ordinal’ package was used specifically for the CLMM analysis within the R environment.

## Results

### Baseline Characteristics

Baseline demographic and clinical characteristics were comparable between the preoperative and postoperative initiation groups, with no significant differences in age, sex distribution, body mass index, operated side, or primary diagnosis (Table 1).

**Table 1:** Baseline characteristics of participants

	Preoperative application initiation group (*n*=76)	Postoperative application initiation group (*n*=61)	95% CI	*p* value
Age (years)	65.0±8.2 (42~83)	66.1±10.6(30~84)	-2.3~4.0	0.59
Sex				
Female	64	50	0.3~2.3	0.85
Male	12	11		
Body mass index (kg/m^2^)	25.2±4.4 (17.7~40.7)	25.1±4.5 (15.9~41.2)	-1.6~1.4	0.89
Primary diagnosis				
Osteoarthritis	74	56	-	0.30
Rapidly destructive coxarthrosis	2	4		
Idiopathic osteonecrosis of the femoral head	0	1		

Values are presented as mean ± standard deviation (range) or number of participants

*CI* confidence interval

### Primary outcome: JHEQ dissatisfaction score

**Fig. 3:**
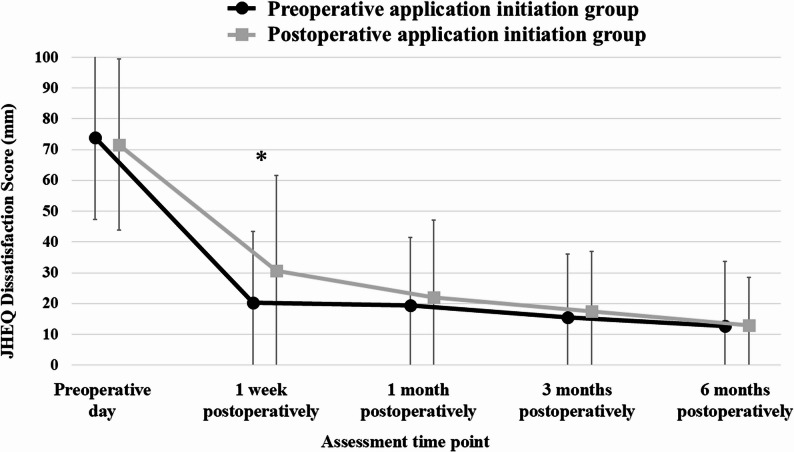
JHEQ dissatisfaction score. Y-axis: JHEQ Dissatisfaction Score (mm), X-axis: Assessment time point. Values are presented as mean ± standard deviation. *p = 0.03 (unadjusted). After Bonferroni correction for five time points, the adjusted *p*-value was 0.15. Data points for each group are slightly offset horizontally to ensure the clarity of error bars at each time point

For JHEQ dissatisfaction scores, a two-way repeated-measures ANOVA revealed a significant interaction between group and time (*p* = 0.03 after Greenhouse-Geisser correction), indicating that the pattern of change over time differed between the two groups. The numerical outcomes (mean ± standard deviation) for the preoperative and postoperative initiation groups were as follows preoperative (74.0 ± 26.6 versus 71.6 ± 27.8), 1 week (20.3 ± 23.1 versus 30.7 ± 30.8), 1 month (19.5 ± 22.1 versus 22.1 ± 25.1), 3 months (15.5 ± 20.5 versus 17.5 ± 19.4), and 6 months postoperatively (12.8 ± 20.9 versus 13.0 ± 15.4). Post-hoc analysis demonstrated that the JHEQ dissatisfaction score at one week postoperatively was lower in the preoperative initiation group than in the postoperative initiation group (mean difference: -10.4, 95% CI: -19.62 to -1.20; unadjusted *p* = 0.03). However, after Bonferroni correction for five time points, this difference was not statistically significant (adjusted *p* = 0.15). The effect size at one week was Cohen’s d = 0.39 (95% CI: 0.04 to 0.74), indicating a small effect with a consistent direction favoring the preoperative group. No significant between-group differences were observed at other time points (Fig. 3).

### Secondary outcomes: FJS-12 and behavioral change stage

Regarding the FJS-12 scores, repeated-measures ANOVA showed a significant main effect of time (*p* < 0.001), but no significant group-time interaction (*p* = 0.53), indicating similar improvement trajectories in both groups. No significant between-group differences were observed at any specific time point.

**Fig. 4:**
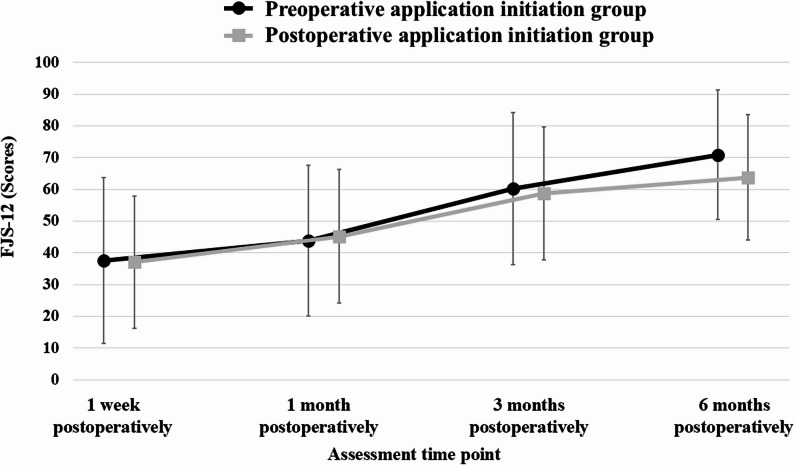
FJS-12. Y-axis: FJS-12 (Scores), X-axis: Assessment time point. Values are presented as mean ± standard deviation. Data points for each group are slightly offset horizontally to ensure the clarity of error bars at each time point

**Fig. 5:**
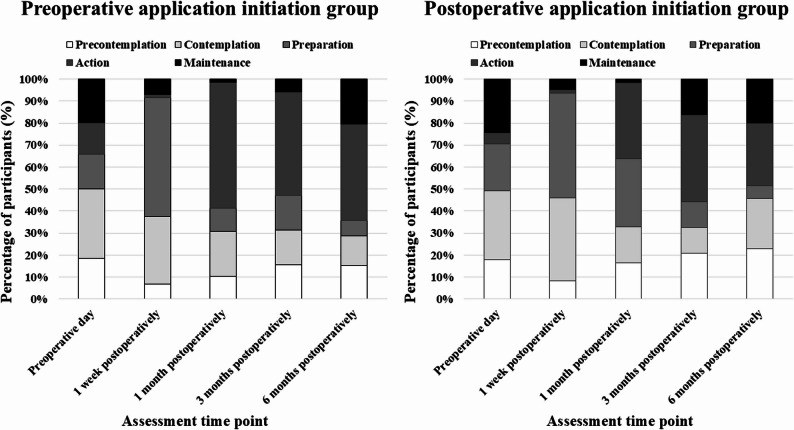
Stages of behavioral change. Changes in stages of behavioral change during the perioperative period. Y-axis: Percentage of participants (%), X-axis: Assessment time point. Distribution of patients across stages of behavioral change (precontemplation, contemplation, preparation, action, and maintenance) in the preoperative application initiation group and the postoperative application initiation group at each assessment time point. Significant between-group differences were observed at the one month postoperatively

For the behavioral change stage, we employed a CLMM to account for the ordinal nature of the data and repeated measurements. While the CLMM did not show a significant group-time interaction (*p* = 0.44), a higher proportion of patients in the preoperative initiation group were classified in the action or maintenance stages at one month postoperatively (*p* = 0.02). No significant differences were observed at other assessment points (Figs. 4 and 5).

## Discussion

The present study demonstrated that preoperative initiation of a smartphone-based physiotherapy support application was associated with clinically relevant reduction in patient dissatisfaction at one week after THA compared with postoperative initiation. While this difference did not reach statistical significance after the strict Bonferroni correction for multiple comparisons (adjusted *p* = 0.15), the observed effect size (Cohen’s d = 0.39) and the fact that the 95% confidence interval for the mean difference (-19.62 to -1.20) did not cross zero suggest a clinically meaningful impact during the early postoperative period. This finding suggests that the timing of delivering perioperative guidance via digital health platforms plays a critical role in shaping the early postoperative patient experience. It should be emphasized that the observed benefits likely reflect the effect of earlier exposure to preoperative education and guidance, which was facilitated by the digital platform, rather than the intrinsic superiority of the smartphone application itself.

Regarding the longitudinal trajectory of patient-reported outcomes, a previous study by Tanaka et al. [18] reported that the JHEQ dissatisfaction VAS improved from 87.06 ± 11.76 preoperatively to 9.78 ± 12.71 at six months after THA. In the present study, the dissatisfaction scores in both groups followed a similar trajectory and eventually reached levels comparable to these reported values by six months. However, our findings highlight that the most pronounced disparity between the two groups occurred in the immediate recovery phase (one week). This suggests that while long-term outcomes eventually equalize, the quality of the initial transition is influenced by the preoperative timing of intervention initiation.

Recent evidence, including a systematic review and meta-analysis by Punnoose A et al. [19], has established that prehabilitation for patients undergoing orthopedic surgery is effective in improving health-related quality of life (HRQOL) and accelerating the recovery of muscle strength. While the efficacy of traditional face-to-face prehabilitation is well-documented, our results suggest that a smartphone-based approach may represent a feasible and scalable option for ensuring early and consistent delivery of this preoperative education. Digital interventions overcome common barriers associated with conventional programs, such as geographical constraints and limited therapist availability. Furthermore, the application serves as a tool for repetitive access to standardized content, which may more effectively enhance patient self-efficacy starting from the moment the surgical decision is made.

Early postoperative dissatisfaction is often driven by pain, anxiety, and uncertainty regarding recovery expectations [4]. Preoperative exposure to structured educational content and exercise guidance through the application likely reduced this uncertainty. Importantly, since the disparity between groups was most evident only at one week postoperatively and was not sustained thereafter, this effect likely reflects improved early patient reassurance and expectation management rather than a sustained improvement in physical function. By familiarizing themselves with the rehabilitation process earlier through the digital platform, patients in the preoperative initiation group likely developed more realistic expectations and better coping mechanisms for the immediate postoperative challenges. These findings are consistent with previous reports indicating that preoperative education can improve early postoperative satisfaction [20, 21].

This pattern further suggests that preoperative digital support primarily influences patients’ initial perception and psychological adaptation during the immediate recovery phase. As postoperative recovery progresses and physical function improves through standardized rehabilitation, the relative contribution of early preoperative education to overall satisfaction may diminish. This is plausible, as the physical benefits of the surgery itself and subsequent recovery eventually become the dominant factors in patient satisfaction. Therefore, our results should be interpreted as a potential improvement in the quality of the initial transition facilitated by psychological preparedness based on the observed effect size and confidence intervals, rather than an enhancement of ultimate clinical or functional outcomes.

Although no significant differences were observed in joint awareness as measured by the FJS-12, the preoperative initiation group demonstrated more favorable behavioral change stages at one month postoperatively. This suggests that while physical joint awareness is largely dictated by the surgical procedure, the emotional and behavioral response can be modulated by the timing of preoperative preparation. This aligns with behavioral theory, which posits that early enhancement of readiness and self-efficacy facilitates engagement in health-promoting behaviors [6–8].

From a clinical perspective, the timing of initiation appears to be a key factor. Using a smartphone application to deliver preoperative support represents a low-risk, scalable strategy to improve the early postoperative patient experience without increasing resource utilization. These findings support the clinical utility of integrating preoperative-start digital health interventions into perioperative rehabilitation pathways for THA, as the magnitude of the effect at one week suggests a consistent benefit in bridging the gap between preoperative anxiety and early postoperative recovery.

### Limitations

This study has several limitations. First, the observational design without random assignment introduces potential selection bias. Allocation was influenced by smartphone ownership and patient preference; thus, patients in the preoperative group may have had higher baseline motivation or health literacy, which could influence postoperative satisfaction.

Second, while repeated-measures analyses were employed to evaluate the overall recovery trajectory, the most prominent between-group differences were observed during the early postoperative phase. Thus, our interpretation of the benefits of preoperative initiation is primarily focused on this critical transition period. Third, application usage intensity (e.g., login frequency) was not quantitatively tracked. Consequently, we cannot determine a dose-response relationship or distinguish whether outcomes were attributable to the intervention itself or to the aforementioned selection bias. Future studies should incorporate digital usage logs to address these points. Fourth, the single-center design and the absence of data on patients’ educational levels or baseline information technology literacy may limit the generalizability of our findings. Fifth, the optimal duration of preoperative application use remains unclear, as patients who started only one day before surgery were excluded. Sixth, it is crucial to distinguish between the effect of the digital platform itself and the effect of earlier exposure to education. The observed benefits likely reflect temporary improvements in reassurance and expectation management-facilitated by the digital tool-rather than sustained functional recovery. Therefore, overinterpretation of these results as evidence of long-term clinical superiority should be avoided.

Finally, this study did not include follow-up beyond six months. Future research should investigate whether early psychological benefits are sustained longer and incorporate sociodemographic variables to clarify how digital tools impact the overall recovery trajectory.

## Conclusions

Earlier (preoperative) initiation of a smartphone-based physiotherapy support application was associated with a notable reduction in patient dissatisfaction at one week after THA, characterized by a clinically relevant effect size (Cohen’s d = 0.39) despite the lack of statistical significance after multiple comparison adjustment. Furthermore, the preoperative initiation group demonstrated more favorable early behavioral readiness for rehabilitation. Shifting the timing of delivering digital health support to the preoperative phase, thereby facilitating earlier exposure to education and rehabilitation guidance, may enhance the early postoperative patient experience and facilitate engagement. Even if the physical outcomes eventually equalize, the preoperative timing of intervention initiation through a digital platform represents a key factor in optimizing the initial transition from hospital to home, as evidenced by the consistent direction of effect observed in the early recovery period.

## Data Availability

The datasets generated and analysed during the current study are not publicly available due to institutional restrictions regarding patient privacy but are available from the corresponding author on reasonable request.

## Abbreviations

THA: Total hip arthroplasty
JHEQ: Japanese Orthopaedic Association Hip Disease Evaluation Questionnaire
FJS-12: Forgotten Joint Score-12
VAS: Visual Analogue Scale
CI: Confidence interval
ANOVA: Analysis of variance
CLMM: Cumulative link mixed model
PROM: Patient-reported outcome measure
HRQOL: Health-related quality of life
